# 2-(2-Hydroxy­ethyl)phthalazin-1(2*H*)-one

**DOI:** 10.1107/S1600536808007691

**Published:** 2008-03-29

**Authors:** Orhan Büyükgüngör, Mustafa Odabaşoğlu

**Affiliations:** a Department of Physics, Faculty of Arts and Sciences, Ondokuz Mayıs University, TR-55139 Kurupelit Samsun, Turkey; b Department of Chemistry, Faculty of Arts and Sciences, Ondokuz Mayıs University, TR-55139 Kurupelit Samsun, Turkey

## Abstract

In the mol­ecule of the title compound, C_10_H_10_N_2_O_2_, the rings are nearly coplanar, making a dihedral angle of 2.35 (5)°. In the crystal structure, inter­molecular C—H⋯O, C—H⋯N and O—H⋯O hydrogen bonds link the mol­ecules, generating *R*_4_^4^(22) and *R*_4_^4^(24) ring motifs to form a three-dimensional network. A weak π–π inter­action between the pyridazinone and benzene rings further stabilizes the crystal structure, with a centroid–centroid distance of 3.709 (3) Å and an inter­planar separation of 3.312 Å.

## Related literature

For general background, see: Cheng *et al.* (1999 ▶); Smith (2001 ▶); Dantzer *et al.* (1999 ▶). For bond-length data, see: Allen *et al.* (1987 ▶). For a related structure, see: Büyükgüngör *et al.* (2007 ▶). For ring motif details, see: Etter (1990 ▶); Bernstein *et al.* (1995 ▶).
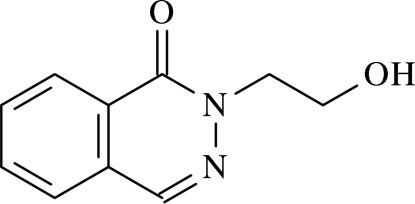


## Experimental

### 

#### Crystal data

C_10_H_10_N_2_O_2_
*M* *_r_* = 190.20
Orthorhombic, 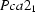

*a* = 7.3278 (6) Å
*b* = 8.1823 (8) Å
*c* = 15.4108 (19) Å
*V* = 924.00 (16) Å^3^
*Z* = 4
Mo *K*α radiation
μ = 0.10 mm^−1^
*T* = 296 K
0.76 × 0.45 × 0.21 mm

#### Data collection

Stoe IPDSII diffractometer
Absorption correction: integration (*X-RED32*; Stoe & Cie, 2002 ▶) *T*_min_ = 0.964, *T*_max_ = 0.982
4205 measured reflections
944 independent reflections
720 reflections with *I* > 2σ(*I*)
*R*_int_ = 0.067

#### Refinement

*R*[*F* ^2^ > 2σ(*F* ^2^)] = 0.089
*wR*(*F* ^2^) = 0.240
*S* = 1.90
944 reflections
98 parameters
1 restraint
H-atom parameters constrained
Δρ_max_ = 0.43 e Å^−3^
Δρ_min_ = −0.40 e Å^−3^

### 

Data collection: *X-AREA* (Stoe & Cie, 2002 ▶); cell refinement: *X-AREA*; data reduction: *X-RED32* (Stoe & Cie, 2002 ▶); program(s) used to solve structure: *SHELXS97* (Sheldrick, 2008 ▶); program(s) used to refine structure: *SHELXL97* (Sheldrick, 2008 ▶); molecular graphics: *ORTEP-3 for Windows* (Farrugia, 1997 ▶); software used to prepare material for publication: *WinGX* (Farrugia, 1999 ▶).

## Supplementary Material

Crystal structure: contains datablocks I. DOI: 10.1107/S1600536808007691/hk2435sup1.cif

Structure factors: contains datablocks I. DOI: 10.1107/S1600536808007691/hk2435Isup2.hkl

Additional supplementary materials:  crystallographic information; 3D view; checkCIF report

## Figures and Tables

**Table 1:** Hydrogen-bond geometry (Å, °)

*D*—H⋯*A*	*D*—H	H⋯*A*	*D*⋯*A*	*D*—H⋯*A*
O2—H2⋯O1^i^	0.82	1.91	2.704 (9)	163
C4—H4⋯N2^ii^	0.93	2.73	3.570 (10)	151
C8—H8⋯O2^iii^	0.93	2.53	3.376 (11)	152

Symmetry codes: (i) 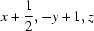; (ii) 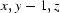; (iii) 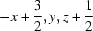.

**Fig. 1.**
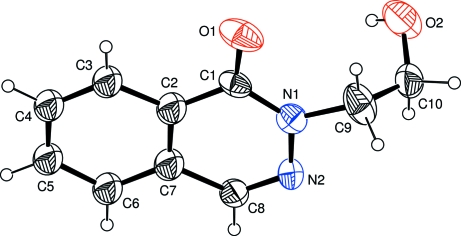
The molecular structure of the title molecule, with the atom-numbering scheme. Displacement ellipsoids are drawn at the 30% probability level.

**Fig. 2.**
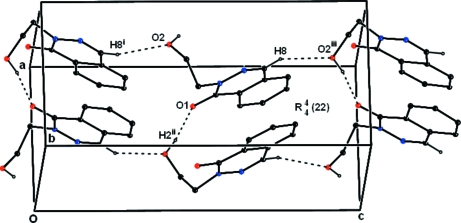
A partial packing diagram of (I), showing the formation of R44(22) ring motifs. Hydrogen bonds are shown as dashed lines [symmetry codes: (i) 3/2 - x, y, z - 1/2; (ii) x - 1/2, 1 - y, z; (iii) 3/2 - x, y, 1/2 + z]. H atoms not involved in hydrogen bondings have been omitted for clarity.

**Fig. 3.**
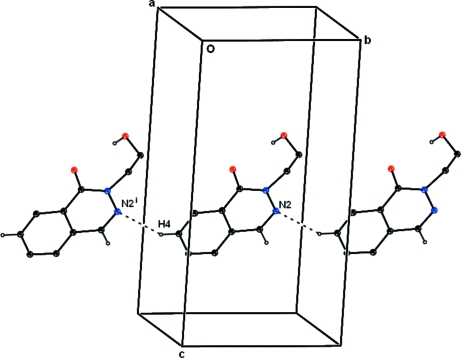
A partial packing diagram of (I), showing the formation of C(7) chain [symmetry code: (i) x, y - 1, z]. H atoms not involved in hydrogen bondings have been omitted for clarity.

**Fig. 4.**
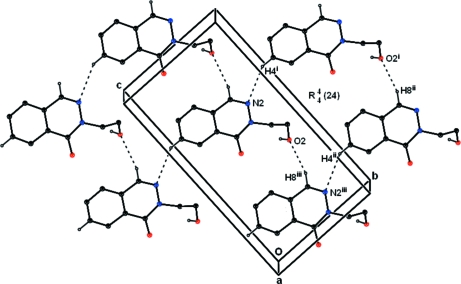
A partial packing diagram of (I), showing the formation of R44(24) ring motifs. Hydrogen bonds are shown as dashed lines [symmetry codes: (i) x, y + 1, z; (ii) 3/2 - x, y + 1, z - 1/2; (iii) 3/2 - x, y, z - 1/2]. H atoms not involved in hydrogen bondings have been omitted for clarity.

**Fig. 5.**
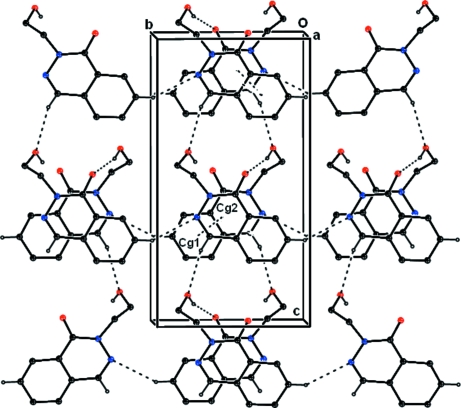
A packing diagram of (I), showing the π···π interactions [symmetry code: (i) x, y - 1, z]. Cg1 and Cg2 denote the centroids of the rings. Hydrogen bonds are shown as dashed lines. H atoms not involved in hydrogen bondings have been omitted for clarity.

## supplementary crystallographic information

### Comment

Phthalazines, also called benzo-*ortho*-diazines or benzopyridazines, are a group of heterocyclic compounds, isomeric with the cinnolines. The practical interest upon phthalazine derivatives is based on their widespread applications. Benzopyridazines, like other members of the isomeric diazene series, have found wide applications such as therapeutic agents, ligands in transition metal catalysis, chemiluminescent and optical materials (Cheng *et al.*, 1999). 2-Substituted-8-(4,6-dimethoxypyrimidin-2-yloxy)-4-methylphthalazine-1-one derivatives are used as herbicides and imide-substituted-4-Benzyl-(2*H*) -phthalazin-1-ones are used as potent inhibitors of poly (ADP-ribose) polymerase-1 (PARP-1) (Smith, 2001; Dantzer *et al.*, 1999). In view of the importance of the phthalazines, we herein report herein the crystal structure of the title compound, (I).

In the molecule of (I), (Fig. 1), the bond lengths (Allen *et al.*, 1987) and angles are within normal ranges (Büyükgüngör *et al.*, 2007). The homoaromatic and heterocyclic rings are, of course, planar and they are also nearly coplanar with a dihedral angle of 2.35 (5)°.

In the crystal structure, intermolecular C-H···O, C-H···N and O-H···O hydrogen bonds (Table 1) link the molecules, generating R_4_^4^(22) (Fig. 2) and R_4_^4^(24) (Fig. 4) ring motifs by C(7) chains (Fig. 3) (Bernstein *et al.*,  1995; Etter, 1990), to form a three-dimensional network, in which they may be effective in the stabilization of the structure. A weak π···π interaction between the pyridazinone and benzene rings, at x, y, z and x - 1/2, 1 - y, z, respectively, further stabilizes the structure, with a centroid-centroid distance of 3.709 (3) Å and plane-plane separation of 3.312 Å (Fig. 5).

### Experimental

A solution of phthalaldehydic acid (1.50 g, 10 mmol) and 3-aminopropan-1-ol (1.52 g, 20 mmol) in DMF (500 ml) was refluxed for 3 h. Crystals of (I) suitable for X-ray analysis were obtained by slow evaporation of a reaction mixture at room temperature (yield; 90%).

### Refinement

H atoms were positioned geometrically, with O-H = 0.82 Å (for OH) and C-H = 0.93 and 0.97 Å for aromatic and methylene H, respectively, and constrained to ride on their parent atoms with U_iso_(H) = xU_eq_(C,O), where x = 1.5 for OH H and x = 1.2 for all other H atoms.

### Crystal data

**Table:**

C_10_H_10_N_2_O_2_	*F*_000_ = 400
*M**_r_* = 190.20	*D*_x_ = 1.367 Mg m^−^^3^
Orthorhombic, *P**c**a*2_1_	Mo *K*α radiation λ = 0.71073 Å
Hall symbol: P 2c -2ac	Cell parameters from 4205 reflections
*a* = 7.3278 (6) Å	θ = 2.5–27.8º
*b* = 8.1823 (8) Å	µ = 0.10 mm^−^^1^
*c* = 15.4108 (19) Å	*T* = 296 K
*V* = 924.00 (16) Å^3^	Prism, colorless
*Z* = 4	0.76 × 0.45 × 0.21 mm

### Data collection

**Table:**

Stoe IPDS II diffractometer	944 independent reflections
Monochromator: plane graphite	720 reflections with *I* > 2σ(*I*)
Detector resolution: 6.67 pixels mm^-1^	*R*_int_ = 0.067
*T* = 296 K	θ_max_ = 26.0º
w–scan rotation method	θ_min_ = 2.5º
Absorption correction: integration(X-RED32; Stoe & Cie, 2002)	*h* = −8→8
*T*_min_ = 0.964, *T*_max_ = 0.982	*k* = −10→9
4205 measured reflections	*l* = −18→18

### Refinement

**Table:**

Refinement on *F*^2^	Secondary atom site location: difference Fourier map
Least-squares matrix: full	Hydrogen site location: inferred from neighbouring sites
*R*[*F*^2^ > 2σ(*F*^2^)] = 0.089	H-atom parameters constrained
*wR*(*F*^2^) = 0.240	*w* = 1/[σ^2^(*F*_o_^2^) + (0.074*P*)^2^ + 0.0928*P*] where *P* = (*F*_o_^2^ + 2*F*_c_^2^)/3
*S* = 1.90	(Δ/σ)_max_ < 0.001
944 reflections	Δρ_max_ = 0.43 e Å^−^^3^
98 parameters	Δρ_min_ = −0.40 e Å^−^^3^
1 restraint	Extinction correction: none
Primary atom site location: structure-invariant direct methods	

### Special details

**Table:**

Geometry. All e.s.d.'s (except the e.s.d. in the dihedral angle between two l.s. planes) are estimated using the full covariance matrix. The cell e.s.d.'s are taken into account individually in the estimation of e.s.d.'s in distances, angles and torsion angles; correlations between e.s.d.'s in cell parameters are only used when they are defined by crystal symmetry. An approximate (isotropic) treatment of cell e.s.d.'s is used for estimating e.s.d.'s involving l.s. planes.
Refinement. Refinement of F^2^ against ALL reflections. The weighted R-factor wR and goodness of fit S are based on F^2^, conventional R-factors R are based on F, with F set to zero for negative F^2^. The threshold expression of F^2^ > 2sigma(F^2^) is used only for calculating R-factors(gt) etc. and is not relevant to the choice of reflections for refinement. R-factors based on F^2^ are statistically about twice as large as those based on F, and R- factors based on ALL data will be even larger.

### Fractional atomic coordinates and isotropic or equivalent isotropic displacement parameters (Å^2^)

**Table:**

	*x*	*y*	*z*	*U*_iso_*/*U*_eq_	
O1	0.5413 (10)	0.4049 (8)	0.4806 (3)	0.095 (2)	
O2	0.7978 (10)	0.7890 (10)	0.3983 (4)	0.110 (2)	
H2	0.8754	0.7473	0.4296	0.166*	
N1	0.5944 (8)	0.6385 (7)	0.5551 (4)	0.0586 (15)	
C1	0.5917 (10)	0.4717 (9)	0.5492 (4)	0.0575 (17)	
C2	0.6416 (14)	0.3841 (8)	0.6254 (6)	0.0726 (10)	
C3	0.6338 (13)	0.2106 (9)	0.6331 (6)	0.0726 (10)	
H3	0.5912	0.1491	0.5866	0.087*	
C4	0.6862 (12)	0.1342 (10)	0.7055 (5)	0.0726 (10)	
H4	0.6827	0.0206	0.7075	0.087*	
C5	0.7455 (13)	0.2200 (9)	0.7774 (6)	0.0726 (10)	
H5	0.7816	0.1640	0.8270	0.087*	
C6	0.7513 (13)	0.3874 (9)	0.7757 (6)	0.0726 (10)	
H6	0.7907	0.4455	0.8240	0.087*	
C7	0.6975 (14)	0.4696 (9)	0.7008 (5)	0.0726 (10)	
C8	0.6945 (12)	0.6446 (8)	0.6931 (5)	0.0624 (19)	
H8	0.7327	0.7041	0.7412	0.075*	
N2	0.6454 (10)	0.7246 (6)	0.6280 (4)	0.0609 (15)	
C9	0.5268 (12)	0.7439 (14)	0.4850 (6)	0.090 (3)	
H9A	0.4774	0.6753	0.4393	0.108*	
H9B	0.4277	0.8103	0.5073	0.108*	
C10	0.6667 (14)	0.8531 (12)	0.4469 (6)	0.092 (3)	
H10A	0.6036	0.9340	0.4120	0.110*	
H10B	0.7248	0.9112	0.4943	0.110*	

### Atomic displacement parameters (Å^2^)

**Table:**

	*U*^11^	*U*^22^	*U*^33^	*U*^12^	*U*^13^	*U*^23^
O1	0.106 (5)	0.124 (5)	0.056 (3)	−0.042 (4)	0.003 (3)	−0.022 (3)
O2	0.098 (5)	0.156 (6)	0.077 (4)	0.036 (5)	0.017 (4)	0.032 (4)
N1	0.048 (3)	0.066 (4)	0.061 (3)	−0.004 (3)	0.003 (3)	0.002 (3)
C1	0.050 (4)	0.073 (4)	0.050 (3)	−0.017 (4)	0.016 (3)	−0.002 (4)
C2	0.079 (2)	0.0575 (16)	0.081 (2)	0.0007 (18)	0.0200 (16)	0.0076 (17)
C3	0.079 (2)	0.0575 (16)	0.081 (2)	0.0007 (18)	0.0200 (16)	0.0076 (17)
C4	0.079 (2)	0.0575 (16)	0.081 (2)	0.0007 (18)	0.0200 (16)	0.0076 (17)
C5	0.079 (2)	0.0575 (16)	0.081 (2)	0.0007 (18)	0.0200 (16)	0.0076 (17)
C6	0.079 (2)	0.0575 (16)	0.081 (2)	0.0007 (18)	0.0200 (16)	0.0076 (17)
C7	0.079 (2)	0.0575 (16)	0.081 (2)	0.0007 (18)	0.0200 (16)	0.0076 (17)
C8	0.085 (5)	0.051 (4)	0.052 (4)	−0.008 (4)	0.003 (3)	−0.004 (3)
N2	0.073 (4)	0.048 (3)	0.062 (3)	−0.002 (3)	0.006 (3)	0.011 (3)
C9	0.063 (5)	0.124 (7)	0.082 (6)	0.013 (5)	−0.007 (5)	0.040 (6)
C10	0.107 (8)	0.084 (5)	0.084 (5)	0.039 (6)	0.027 (5)	0.032 (5)

### Geometric parameters (Å, °)

**Table:**

O2—H2	0.8200	C6—H6	0.9300
C1—O1	1.246 (9)	C7—C8	1.436 (10)
C1—N1	1.368 (9)	C8—N2	1.251 (9)
C1—C2	1.423 (11)	C8—H8	0.9300
C2—C7	1.417 (12)	N2—N1	1.377 (9)
C2—C3	1.426 (10)	C9—C10	1.481 (14)
C3—C4	1.335 (11)	C9—N1	1.469 (10)
C3—H3	0.9300	C9—H9A	0.9700
C4—C5	1.382 (13)	C9—H9B	0.9700
C4—H4	0.9300	C10—O2	1.327 (10)
C5—C6	1.371 (11)	C10—H10A	0.9700
C5—H5	0.9300	C10—H10B	0.9700
C6—C7	1.392 (12)		
			
C10—O2—H2	109.5	C7—C6—H6	120.3
C1—N1—N2	124.7 (6)	C6—C7—C2	121.5 (7)
C1—N1—C9	122.1 (7)	C6—C7—C8	123.7 (8)
N2—N1—C9	113.0 (7)	C2—C7—C8	114.8 (7)
O1—C1—N1	119.9 (7)	N2—C8—C7	126.4 (7)
O1—C1—C2	123.7 (7)	N2—C8—H8	116.8
N1—C1—C2	116.4 (7)	C7—C8—H8	116.8
C7—C2—C3	115.8 (8)	C8—N2—N1	117.6 (5)
C7—C2—C1	120.1 (6)	C10—C9—N1	114.3 (7)
C3—C2—C1	124.0 (9)	C10—C9—H9A	108.7
C4—C3—C2	121.6 (9)	N1—C9—H9A	108.7
C4—C3—H3	119.2	C10—C9—H9B	108.7
C2—C3—H3	119.2	N1—C9—H9B	108.7
C5—C4—C3	121.5 (8)	H9A—C9—H9B	107.6
C5—C4—H4	119.2	O2—C10—C9	119.1 (9)
C3—C4—H4	119.2	O2—C10—H10A	107.5
C6—C5—C4	120.1 (8)	C9—C10—H10A	107.5
C6—C5—H5	119.9	O2—C10—H10B	107.5
C4—C5—H5	119.9	C9—C10—H10B	107.5
C5—C6—C7	119.3 (8)	H10A—C10—H10B	107.0
C5—C6—H6	120.3		
			
C8—N2—N1—C1	0.7 (11)	C4—C5—C6—C7	−0.2 (14)
C8—N2—N1—C9	175.8 (7)	C5—C6—C7—C2	−1.8 (15)
O1—C1—N1—N2	179.2 (6)	C5—C6—C7—C8	178.5 (8)
C2—C1—N1—N2	1.6 (10)	C3—C2—C7—C6	3.6 (14)
O1—C1—N1—C9	4.6 (10)	C1—C2—C7—C6	−178.5 (8)
C2—C1—N1—C9	−173.0 (7)	C3—C2—C7—C8	−176.7 (8)
O1—C1—C2—C7	180.0 (8)	C1—C2—C7—C8	1.3 (13)
N1—C1—C2—C7	−2.5 (12)	C6—C7—C8—N2	−179.1 (9)
O1—C1—C2—C3	−2.2 (13)	C2—C7—C8—N2	1.2 (13)
N1—C1—C2—C3	175.3 (8)	C7—C8—N2—N1	−2.2 (13)
C7—C2—C3—C4	−3.7 (13)	C10—C9—N1—C1	−118.4 (9)
C1—C2—C3—C4	178.4 (8)	C10—C9—N1—N2	66.5 (11)
C2—C3—C4—C5	1.9 (14)	N1—C9—C10—O2	70.9 (12)
C3—C4—C5—C6	0.1 (14)		

### Hydrogen-bond geometry (Å, °)

**Table:**

*D*—H···*A*	*D*—H	H···*A*	*D*···*A*	*D*—H···*A*
O2—H2···O1^i^	0.82	1.91	2.704 (9)	163
C4—H4···N2^ii^	0.93	2.73	3.570 (10)	151
C8—H8···O2^iii^	0.93	2.53	3.376 (11)	152

Symmetry codes: (i) *x*+1/2, −*y*+1, *z*; (ii) *x*, *y*−1, *z*; (iii) −*x*+3/2, *y*, *z*+1/2.
